# Histone Deacetylase Inhibitors Induce Epithelial-to-Mesenchymal Transition in Prostate Cancer Cells

**DOI:** 10.1371/journal.pone.0045045

**Published:** 2012-09-14

**Authors:** Dejuan Kong, Aamir Ahmad, Bin Bao, Yiwei Li, Sanjeev Banerjee, Fazlul H. Sarkar

**Affiliations:** Department of Pathology, Karmanos Cancer Institute, Wayne State University School of Medicine, Detroit, Michigan, United States of America; University of Alabama at Birmingham, United States of America

## Abstract

Clinical experience of histone deacetylase inhibitors (HDACIs) in patients with solid tumors has been disappointing; however, the molecular mechanism of treatment failure is not known. Therefore, we sought to investigate the molecular mechanism of treatment failure of HDACIs in the present study. We found that HDACIs Trichostatin A (TSA) and Suberoylanilide hydroxamic acid (SAHA) could induce epithelial-to-mesenchymal transition (EMT) phenotype in prostate cancer (PCa) cells, which was associated with changes in cellular morphology consistent with increased expression of transcription factors ZEB1, ZEB2 and Slug, and mesenchymal markers such as vimentin, N-cadherin and Fibronectin. CHIP assay showed acetylation of histone 3 on proximal promoters of selected genes, which was in part responsible for increased expression of EMT markers. Moreover, TSA treatment led to further increase in the expression of Sox2 and Nanog in PCa cells with EMT phenotype, which was associated with cancer stem-like cell (CSLC) characteristics consistent with increased cell motility. Our results suggest that HDACIs alone would lead to tumor aggressiveness, and thus strategies for reverting EMT-phenotype to mesenchymal-to-epithelial transition (MET) phenotype or the reversal of CSLC characteristics prior to the use of HDACIs would be beneficial to realize the value of HDACIs for the treatment of solid tumors especially PCa.

## Introduction

A wide variety of genetic and genomic alterations such as amplifications, translocations, deletions, and point mutations has been believed to be associated with cancer development. However, recent studies have demonstrated that epigenetic changes are also involved in cancer development [1]. The main modifications in humans are DNA methylation and posttranslational histone modifications including acetylation, methylation, phosphorylation, etc, which are involved in deregulated expression of genes mediated by transcriptional regulation [1], [2]. Acetylation and deacetylation of histones plays important roles in the transcriptional regulation of genes in the eukaryotic cells. The status of histone acetylation is dependent on the balance of the activities of histone acetyltransferase (HAT) and histone deacetylase (HDAC). HDACs remove the acetyl groups from lysine in the histone tail, which promotes more condensed chromatin structure, resulting in the repression of gene transcription by limiting the accessibility of the transcription factors [3]. Increased expression and activity of HDACs in cancer tissues led to the rational design of histone deacetylase inhibitors (HDACIs) as potential therapeutic agents for cancer therapy. Several HDACIs have been used in phase I and II clinical trial for the treatment of a number of hematological malignancies and also solid tumors [4]. Most of the positive responses to HDACIs were found to be in patients with hematological malignancies including cutaneous T-cell lymphoma and peripheral T-cell lymphoma. However, the results in solid tumors, thus far, have been disappointing. To date, several mechanisms by which resistance are induced during the treatment of solid tumors with HDACIs have been elucidated, including increased expression of the multidrug-resistance gene, MDR1 (ABCB1), increased anti-apoptotic proteins and activating cell survival pathway [3], and such findings have not yet been translated into clinical medicine. Therefore, better understanding of the molecular determinants of resistance to HDACIs could provide the basis for the development of novel therapeutic strategies that could improve the treatment outcome of patients diagnosed with solid tumors.

Epithelial-to-Mesenchymal Transition (EMT) is believed to be associated with drug-resistance [5], [6]. The biology of EMT is a crucial trans-differentiation process, which occurs during embryogenesis and in adult tissues following wound repair and organ remodeling in response to injury, and also occurs during cancer progression [7], [8]. During this process, the epithelial cells acquire mesenchymal cell morphology through down-regulation of epithelial markers and up-regulation of mesenchymal markers, thereby leads to increased migratory capacity, invasiveness and increased resistance to chemotherapy, and all of which are involved in cancer progression [7]–[15]. Moreover, the cells with EMT phenotype share characteristics with cancer stem-like cell (CSLC), which confers drug resistance to these cells and contributes to cancer recurrence and metastasis [10], [11], [16], [17]. Kong et al., found that over-expression of PDGF-D led to the induction of EMT phenotype in PC3 prostate cancer (PCa) cells, which was associated with loss of epithelial markers and gain of expression of mesenchymal markers such as N-cadherin, vimentin as well as up-regulation of transcription factors including ZEB1, ZEB2 and Slug, resulting in enhanced cell migration, invasion i*n vitro* and tumor growth *in vivo* *[9]*, [18], *[19]*. Furthermore, PDGF-D over-expressing PC3 cells (PC3 PDGF-D cells) with EMT phenotype displayed CSLC signatures, which was consistent with increased expression of Oct4. Nanog, Sox2 and Lin28B, resulting in enhanced clonogenic ability and self-renewal capacity [10].

Klarmann et al. demonstrated that invasive cells through Matrigel underwent EMT phenotypic conversion and displayed increased expression of CD44 and decreased expression of CD24, and increased Hedgehog signaling. Moreover, invasive cells from DU145 cells and cells from human primary PCa are more tumorigenic in NOD/SCID mice compared with non-invasive cells [20]. Mechanistically, the co-operation between RAS activation and loss of PTEN led to the acquisition of stem/progenitor subpopulation with mesenchymal characteristics, and these cells were highly metastatic upon orthotopic transplantation [21]. Low expression of ETS transcription factor ESE3/EHF was demonstrated to be associated with increased biochemical recurrence and reduced overall survival of PCa patients after radical prostatectomy, which was found to be consistent with induction of EMT and acquisition of CSLC signatures that led to tumor-initiating and metastatic properties of PCa cells [22]. Androgen-deprivation therapy (ADT) is commonly used for the treatment of metastatic PCa, and Jennbacken et al. showed that ADT could enhance N-cadherin expression, a hallmark for EMT, which was associated with increased of metastasis [23]. Increased expression of N-cadherin in non-metastatic, androgen-dependent PCa in animal model led to castration resistance, increased invasion and metastasis, which was consistent with findings showing elevated expression of N-cadherin in primary and metastatic tumors of patients with castration resistant PCa (CRPC) [24]. Moreover, a recent study has shown that ADT could induce EMT and leads to the acquisition of CSLC signatures in both normal mouse prostate tissue and human LuCaP35 prostate tumor explants. Similar changes in mesenchymal features were also observed in prostate tumors from patients treated with ADT [25]. These results suggest that the induction of EMT and CSLC signatures leads to therapeutic resistance and leads to prostate cancer progression.

In the present study, we found that treatment of PCa cells with TSA or SAHA induced EMT phenotype mediated through up-regulation of transcription factors and mesenchymal markers via promoting acetylation of histone 3 on proximal promoters of specific genes. Moreover, TSA treatment also led to further increase in the expression of stem cell markers in EMT phenotypic PCa cells. These results were consistent with increased cell motility of TSA or SAHA treated PCa cells. our results provide a mechanistic basis for the disappointing outcome of HDACIs in the treatment of solid tumors, and further suggest that reversal of EMT phenotype or CSLC characteristics by novel approach prior to HDACIs could become a rational strategy for the treatment of solid tumors especially PCa.

**Table 1:** The sequences of primers used in this study.

Primer name	Sequence (5′–3′)	Application
ZEB1-F^A^	GCACAACCAAGTGCAGAAGA	RT-PCR
ZEB1-R	GCCTGGTTCAGGAGAAGATG	RT-PCR
ZEB2-F	CAAGAGGCGCAAACAAGC	RT-PCR
ZEB2-R	GGTTGGCAATACCGTCATCC	RT-PCR
Slug-F	CTACAGCGAACTGGACACACA	RT-PCR
Slug-R	GGAATGGAGCAGCGGTAGT	RT-PCR
Twist-F	GTGGCTCACGAGCGGCTCAG	RT-PCR
Twist-R	CTAGGTCTCCGGCCCTGCTG	RT-PCR
N-cadherin-F	CCTGCGCGTGAAGGTTTGCC	RT-PCR
N-cadherin-F	CCAAGCCCCGCACCCACAAT	RT-PCR
Vimentin-F	AGATGGCCCTTGACATTGAG	RT-PCR
Vimentin-R	TGGAAGAGGCAGAGAAATCC	RT-PCR
MMP2-F	GCTGGCCTAGTGATGATGTTAGGCA	RT-PCR
MMP2-R	CCTTGGGGCAGCCATAGAAGGT	RT-PCR
GAPDH-F	TTCTTTTGCGTCGCCAGCCGA	RT-PCR
GAPDH-R	GTGACCAGGCGCCCAATACGA	RT-PCR
Vimentin-p-F^B^	CGCCCTCGTTCGCCTCTTCT	CHIP assay
Vimentin-p-R	GGACATGGCTGCGGAGGGTG	ChIP assay
ZEB2-P-F	GTTTCAATGGGCGCGGGCGA	CHIP assay
ZEB2-P-F	GGCAGCACGCAGGCTCGAT	ChIP assay
Slug-p-R	GTCCGTCTGCCGCACCTGAG	CHIP assay
Slug-p-R	ACACGGCGGTCCCTACAGCA	ChIP assay
MMP2-p-R	GAGCAGGCTCCAACCAGGCG	CHIP assay
MMP2-p-F	GTAGCGCTCCCTGGCCCCG	ChIP assay

A F: forward primer; R: reverse primer.

B Vimentin-p-F: forward primer for vimentin promoter, Vimentin-p-R: reverse primer for vimentin promoter.

## Materials and Methods

### Cell Lines and Culture Condition

LNCaP and PC3 cells were purchased from American Type Culture Collection (ATCC, Manassas, VA) and maintained in RPMI 1640 (Invitrogen, Carlsbad, CA) supplemented with 10% fetal bovine serum (FBS), 10 µmol/L Hepes, 50 units/ml Penicillin and 50 µg/ml Streptomycin. Stable cell lines over-expressing PDGF-D (referred to PC3 PDGF-D) was generated as previously described [19] and cultured in RPMI 1640 (Invitrogen) supplemented with 5% fetal bovine serum (FBS), 2 mmol/L glutamine, 10 µmol/L Hepes, 50 units/ml Penicillin, and 50 µg/ml Streptomycin. All cells were maintained in a 5% CO_2_-humidified atmosphere at 37°C and genotypically characterized to support the authenticity of these cells, which was consistent with its origin.

### Research Reagents and Antibodies

Trichostatin A (TSA) was obtained from Sigma Aldrich (St. Louis, MO) and Suberoylanilide hydroxamic acid (SAHA) was purchased from Aton Pharma, Inc, (Lawrenceville, NJ). Antibodies against acety-histone H3 (Lys9), HDAC1, HDAC2, HDAC3, HDAC4, HDAC6, Slug, Sox2 and Nanog were purchased from Cell Signaling Technology (Beverly, MA). Antibodies against N-cadherin and vimentin were purchased from BD Biosciences (Bedford, MA) and Abcam (Cambridge MA), respectively. Antibodies against ZEB1, Fibronectin and E-cadherin were obtained from Santa Cruz (Santa Cruz, CA). Goat anti-rabbit IgG (H+L)-HRP conjugate and goat anti-mouse IgG (H+L)-HRP conjugate were purchased from Bio-Rad (Reinach, BL). Antibody to glyceraldehyde 3-phosphate dehydrogenase (GAPDH) was obtained from Affinity BioReagents (Golden, CO). Alex Fluro 594 goat anti-rabbit IgG or Alex Fluro 594 goat anti-mouse IgG and Alexa Fluor 594 phalloidin for F-actin staining were purchased from Invitrogen (Carlsbad, CA).

**Figure 1:**
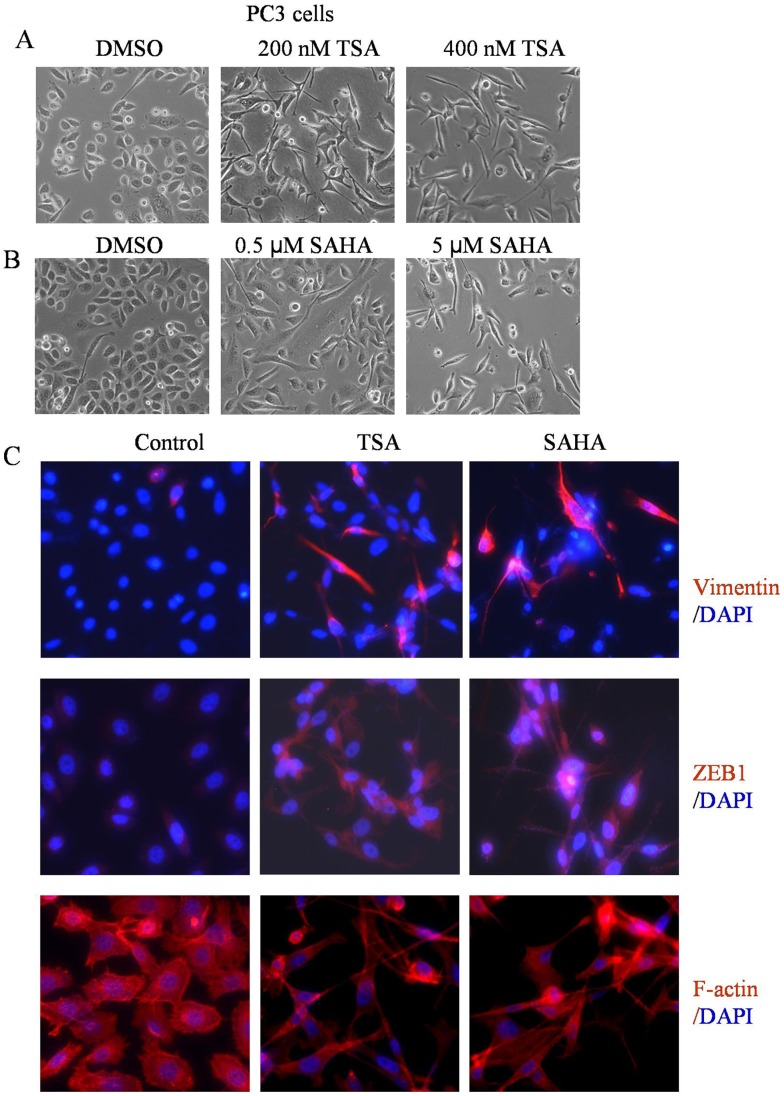
HDACIs led to the induction of EMT phenotype. (A and B) PC3 cells treated with TSA and SAHA for 24 h at indicated doses. The photomicrographs of PC3 cells treated with TSA and SAHA exhibited a fibroblastic-type phenotype, while cells treated with DMAO control displayed rounded epithelial cell morphology (original magnification, ×100). (C) PC3 cells were seeded in the chamber. After 24 h incubation, cells were treated with 400 nM TSA or 5 µM SAHA for another 24 h. The results from immuno-fluorescence staining for vimentin and ZEB1 (Red) indicated that treatment of PC3 cells with TSA and SAHA increased the expression of vimentin and ZEB1 (middle and right panel) compared to control cells (left panel). Phalloidin staining showed F-actin (Red) reorganization in TSA and SAHA treated PC3 cells. Nuclear DNA was stained with DAPI (Blue, original magnification, ×200).

**Figure 2:**
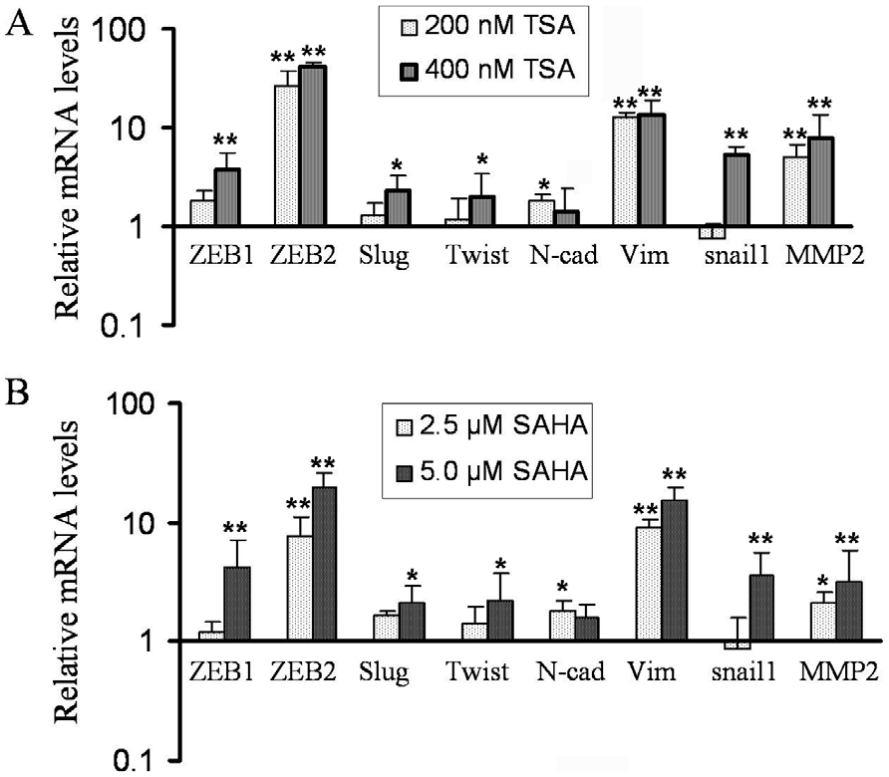
TSA and SAHA increased the expression of EMT-related transcription factors and mesenchymal markers in PCa cells at different doses. Total RNA was isolated from PC3 cells treated with 200 nM and 400 nM TSA (A) or 2.5 µM and 5 µM SAHA (B) for 24 h. The results from real time RT-PCR showed that the relative mRNA expression of ZEB1, ZEB2, Slug, Twist, N-Cadherin, vimentin and MMP2 was increased following treatment compared to DMSO control (the value of control was designed as 1, * p<0.05, ** p<0.01).

### Cell Migration Assay

Cell migration assay was performed using 24 well Transwell Permeable Supports with 8 µm pores (Corning, Lowell, MA) according to the instruction supplied by the manufacturer. Briefly, PC3 cells treated with TSA, SAHA or DMSO control for 6 hours were collected and suspended in serum free RPMI 1640 medium and 1×10^5^ cells in each well were seeded into the Transwell inserts. Bottom wells was filled with 0.5 ml of media containing 10% FBS. After 24 h of incubation, migrated cells were determined as previously described [9].

### Western Blot Analysis

Total cell lysates were prepared by lysing the cells in RIPA buffer. Protein concentrations were measured using protein assay reagents (Pierce, Rockford, IL). Proteins with equal amount were subjected to SDS-PAGE to separate and electrophoretically transferred to nitrocellulose membranes. After blocking with 3% milk, the protein was detected by incubating with respective primary antibodies followed by incubation with respective secondary antibodies as described previously [26].

### Real-time RT-PCR

The total RNA was isolated using RNeasy mini kit (Qiagen, Valencia, CA). The residual DNA was removed using an RNase-free NDase kit (Qiagen). High Capacity RNA-to-cDNA Kit (Applied biosystems, Fostor, CA) was used to reverse transcribed one microgram of RNA into cDNA according to the manufacturer’s instruction. Real time PCR was performed using specific primers to quantify gene expression by using SYBR® Green RT-PCR Reagents (Applied biosystems). The relative amount of mRNA was normalized to the expression of GAPDH. Primer sequences are shown in the table 1.

### CHIP Assay

SimpleChIP® Enzymatic Chromatin IP Kit (Magnetic Beads) and 6-Tube Magnetic Separation Rack were purchased from cell signaling and used for performing CHIP assay according to the manufacturer’s instruction. Briefly, PC3 cells treated with 400 nM TSA and 5 µM SAHA or DMSO control for 16 hours and 37% formaldehyde was added (Final formaldehyde concentration was 1%) to crosslink proteins to DNA and mixed, and then incubated for 10 minutes at room temperature. Glycine was added into dishes and the cells were incubated for 5 minutes at room temperature. After washing with ice-cold PBS, the nuclear extracts from the cells wee prepared. For cross-linked chromatin preparation, the nuclear suspension was added to Micrococcal Nuclease to digest DNA to length of approximately 150–900 bp and Micrococcal Nuclease was inactivated by EDTA, and then sonicated with several pulses to break nuclear membrane. After purification, DNA fragment size was determined by electrophoresis on a 1% agarose gel and results showed that ranges of DNA size was approximately 150–900 bp length. For chromatin immunoprecipitation, 15 µg of chromatin DNA in CHIP buffer (for each immunoprecipitation) was incubated with the immunoprecipitating antibody: Ac-H3, HDAC1, HDAC2, HDAC3, HDAC4, HDAC6, and the negative control (Normal Rabbit IgG) at 4°C with rotation. After incubation overnight, 30 µl of ChIP Grade Protein G Magnetic Beads were added and incubated for 2 h at 4°C with rotation Chromatin was eluted from Antibody/Protein G Beads and cross-links was reversed by incubating with Proteinase K 2 hours at 65°C. DNA was purified by using Spin Columns. Quantification of DNA was performed by PCR using specific primers for gene promoter of vimentin, ZEB2, Slug and MMP2. Primer sequences for these gene promoters are shown in the table 1. The relative amount of promoter DNA was normalized to the input. IgG was used as reference control and calculated as unit value of 1.0.

**Figure 3:**
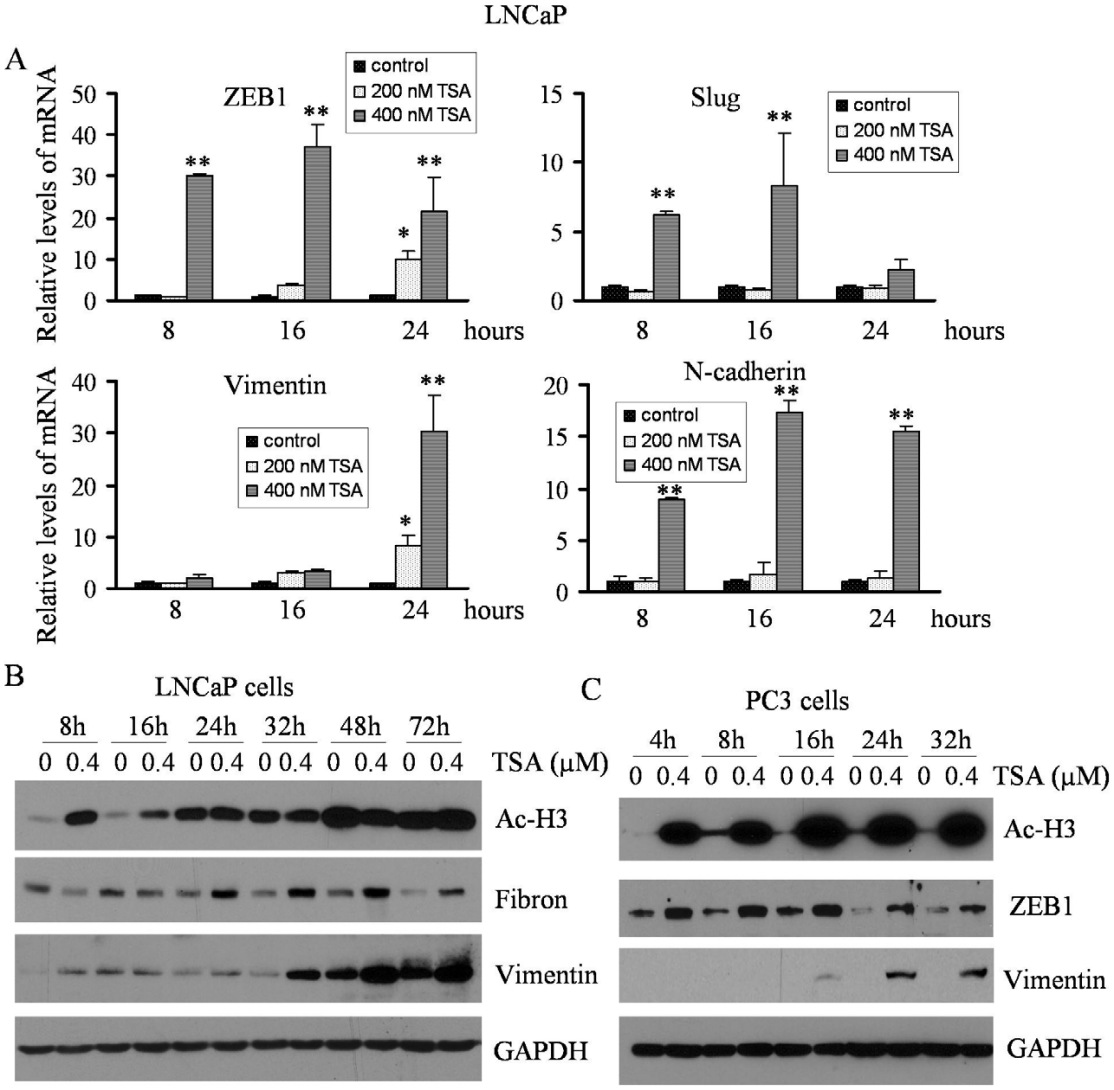
TSA treatment increased the expression of EMT-related factors with concomitant hyper-acetylation of histone 3 at different time points. (A) Total RNA was isolated from LNCaP cells treated with 200 nM and 400 nM TSA for 8, 16 and 24 h. The relative mRNA expression of ZEB1, Slug, vimentin and N-cadherin was increased following TSA treatment compared with DMSO control (the value of control was designed as 1, *, p<0.05; **, p<0.01). (B) LNCaP cells were treated with 400 nM TSA for 8 h to 72 h. Western blot analysis showing that TSA treatment promoted acetylation of histone3 (Ac-H3) at 8 h and 16 h treatment. The expression of mesenchymal markers such as Fibronectin (Fibron) and vimentin was elevated after 24 h treatment with TSA. (C) PC3 cells were treated with 400 nM TSA for 4 h to 32 h. Hyper-acetylation of histone 3 was seen at 4 h to 32 h treatment with concomitant increased expression of ZEB1 in each time point of treatment. Enhanced expression of vimentin was observed after 16 h of treatment with TSA.

**Figure 4:**
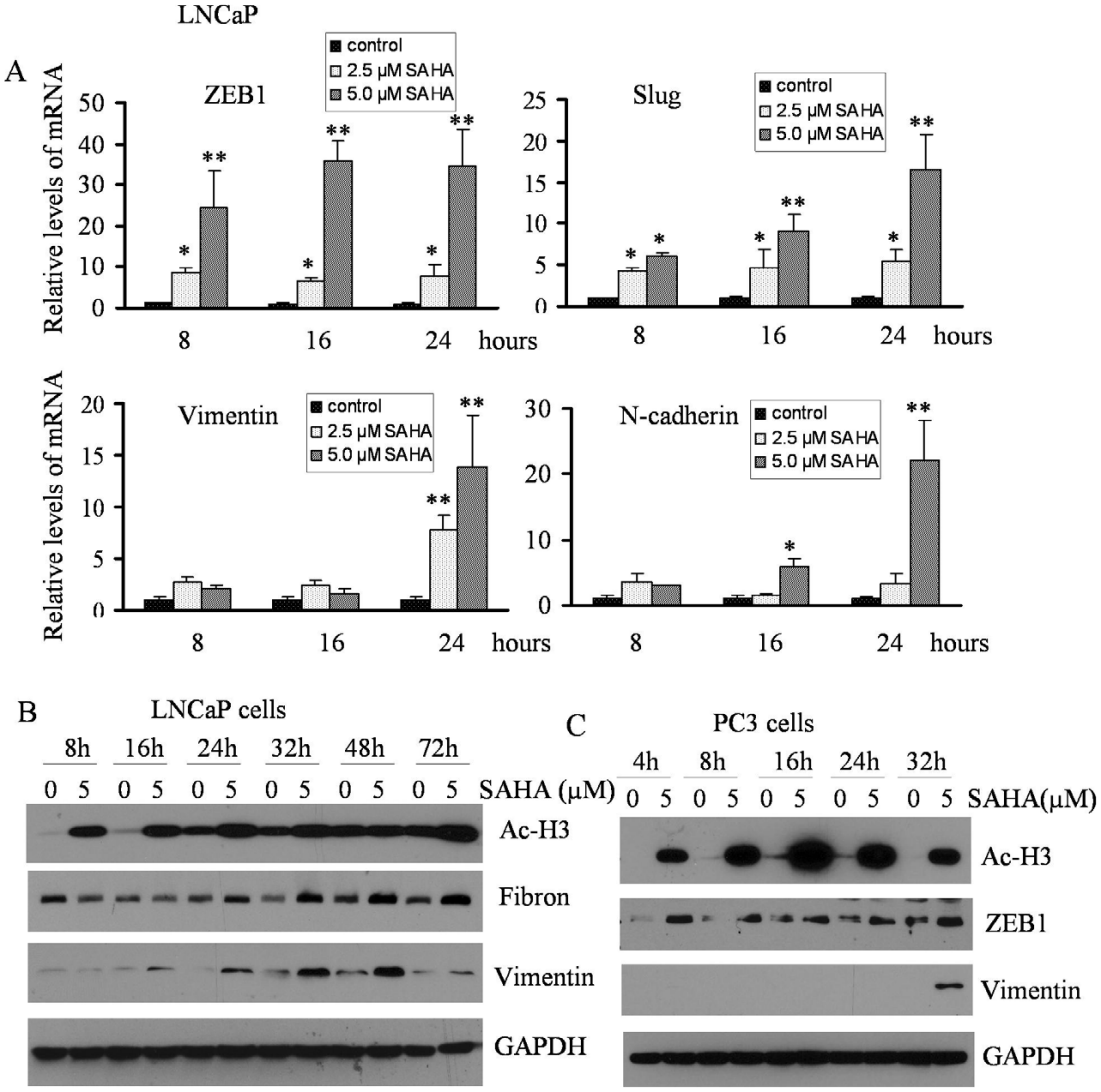
SAHA treatment promoted the expression of EMT-related factors and acetylation of histone 3 at different time points. (A) Total RNA was isolated from LNCaP cells treated with 2.5 µM and 5 µM of SAHA for 8, 16 and 24 h. The results from real time RT-PCR showed that the relative mRNA expression of transcription factors such as ZEB1 and Slug was up-regulated as early as 8 h of treatment with SAHA, while vimentin and N-Cadherin was increased after 16 h of treatment compared to DMSO control (the value of control was designed as 1, *, p<0.05; **, p<0.01). (B) LNCaP cells were treated with 5 µM SAHA for 8 h to 72 h. Western blot analysis showing that SAHA treatment promoted acetylation of histone3 (Ac-H3) at 8 h to 72 h treatment. Fibronectin (Fibron) significantly increased after 24 h of treatment and vimentin was dramatically elevated after 16 h of treatment with SAHA. (C) PC3 cells were treated with 5 µM SAHA for 4 h to 32 h. and increased expression of ZEB1 was observed at 4 h to 32 h treatment, which was associated with Hyper-acetylation of histone 3. Enhanced expression of vimentin was observed at 32 h of treatment with SAHA.

### Immuno-fluorescence Microscopy

Immuno-fluorescence staining was performed as described previously by our laboratory [18]. Briefly, cells were fixed with 4% paraformaldehyde for 15 minutes at room temperature (RT). After washing 3 times with PBS, the cells were permeabilized in 0.5% Triton x-100 in PBS for 10 minutes, and then blocked with 10% goat serum. The cells were incubated for 40 minutes with antibodies against vimentin (pre-diluted) and ZEB1 (1∶50) in 5% goat serum, washed and then incubated for another 40 minutes with Alex Fluro 594 conjugated secondary antibody (1∶250) at room temperature. For F-actin staining, the cells were fixed with 4% paraformaldehyde for 15 minutes and permeabilized in 0.5% Triton x-100 in PBS for 10 minutes, and then incubated with 0.33 µM of Alexa Fluor 594 phalloidin for 40 minutes at 4°C. After washing, the slides were mounted with mounting medium containing anti-fade reagent and DAPI. The cells were viewed under fluorescence microscope. The images were captured and analyzed by using Advanced Sport software (Diagnostic Instruments, Sterling Heights, MI).

### Cell Detachment Assay

For cell detachment assay, the cells were seeded in 24-well plates at 5×10^4^ cells per well. After 8 h incubation, the cells were treated with 400 nM TSA and 5 µM SAHA or DMSO control for 20 hours. For detaching the cells from the culture plates, the medium was removed and the cells were washed with PBS and incubated with 0.125% trypsin EDTA at RT for 3 minutes. After inactivating the trypsin by adding the medium containing 5% FBS into the wells, the detached cells were collected into tubes. The remaining cells were incubated with 0.25% trypsin EDTA for 5 minutes at 37°C to detach all the cells from the culture plates and the cell suspensions were collected into new tubes. The cells were counted and the data were presented as a percentage of the detached cells (incubation with 0.125% trypsin EDTA) to total cells.

### Data Analysis

Experiments were performed in three or more different repetitions. The data was presented as the mean values ± SE. A two-tailed student’s *t* test was used for comparisons between the two groups. ANOVA was used for comparisons of more than two groups. Values of p<0.05 were considered to be statistically significant.

## Results

### HDACIs Induced EMT Phenotype in Prostate Cancer Cells

To investigate the effects of HDACIs on cell morphology, we treated PC3 cells with TSA or SAHA, and we found that PC3 cells treated with TSA or SAHA displayed elongated/irregular fibroblastoid morphology. In contrast, PC3 cells treated with DMSO exhibited a cobblestone appearance, typical morphology of epithelial cells (Fig. 1A and B). DU145 and ARCaPE cells treated with TSA or SAHA also displayed elongated/irregular fibroblastoid morphology (Data not shown). These results suggest that treatment of prostate cancer cells with HDACIs leads to the induction of EMT phenotype. To further confirm whether HDACIs could really induce EMT in PC3 cells, we determined the expression of vimentin and ZEB1 using immuno-fluorescence staining. TSA and SAHA induced EMT phenotype was consistent with increased expression of vimentin and ZEB1 (Fig. 1C, upper and middle panel). In addition, F-actin reorganization and a diffuse pattern were also observed in PC3 cells treated with TSA and SAHA (Fig. 1C, lower panel), which were correlated with EMT phenotype.

**Figure 5:**
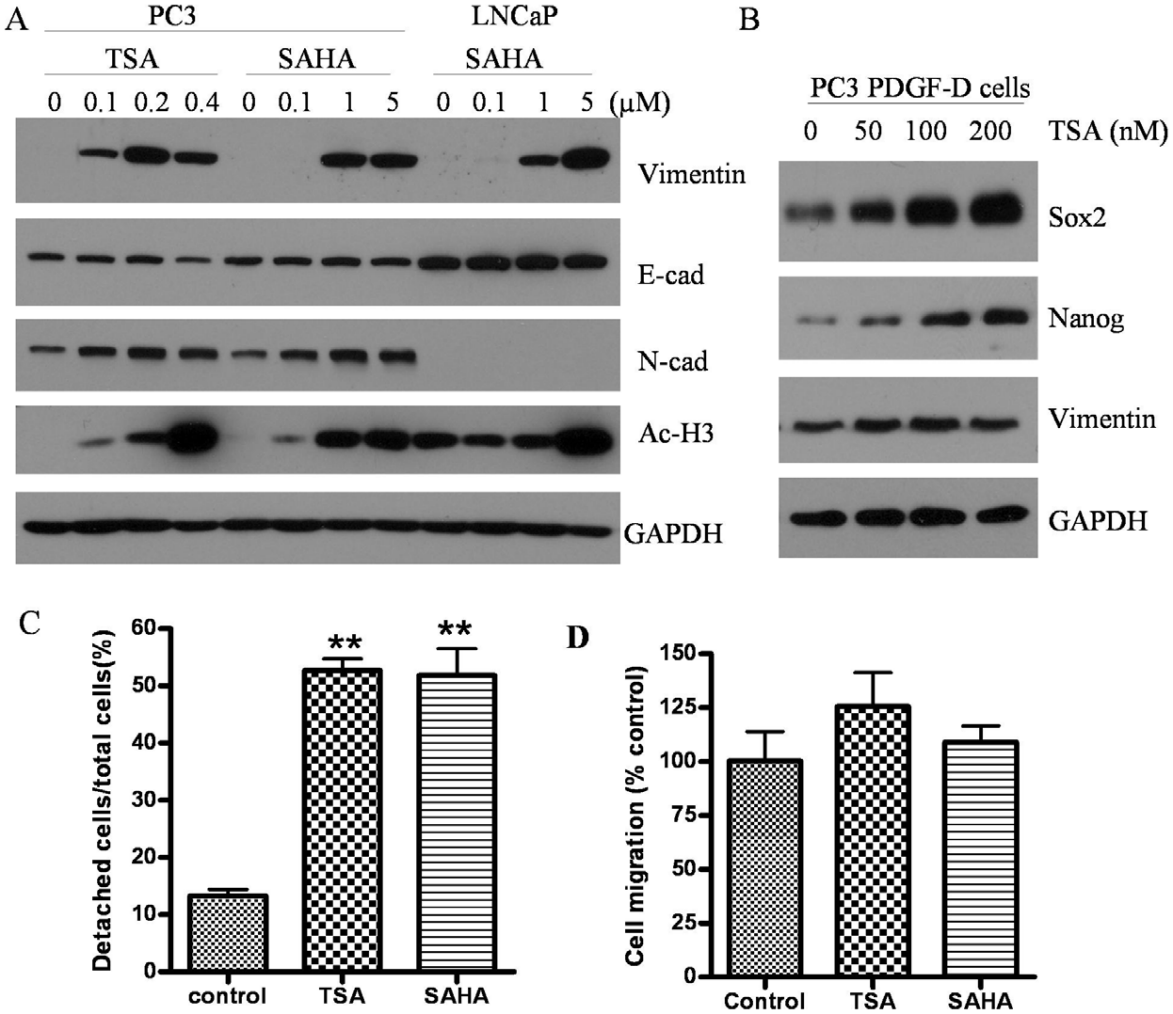
HDACIs not only induced EMT but also increased the expression of cancer stem cell markers associated with increased cell motility. (A) PC3 and LNCaP cells were treated with TSA or SAHA at different doses for 48 h. Western blot analysis showing the expression of epithelial and meshenchymal markers as well as acetylating status. (B) TSA treatment for 48 h increased the expression of Sox2 and Nanog in a dose dependent manner in PC3 PDGF-D cells. Up-regulation of vimentin was seen even after 50 nM TSA of treatment. (C) Cell detachment assay was performed after 400 nM TSA or 5 µM SAHA treatment for 20 h. TSA and SAHA significantly promoted PC3 cell detachment from culture surfaces (**, p<0.01). (D) Showing increased trends of cell migration of PC3 cells treated with TSA and SAHA.

**Figure 6:**
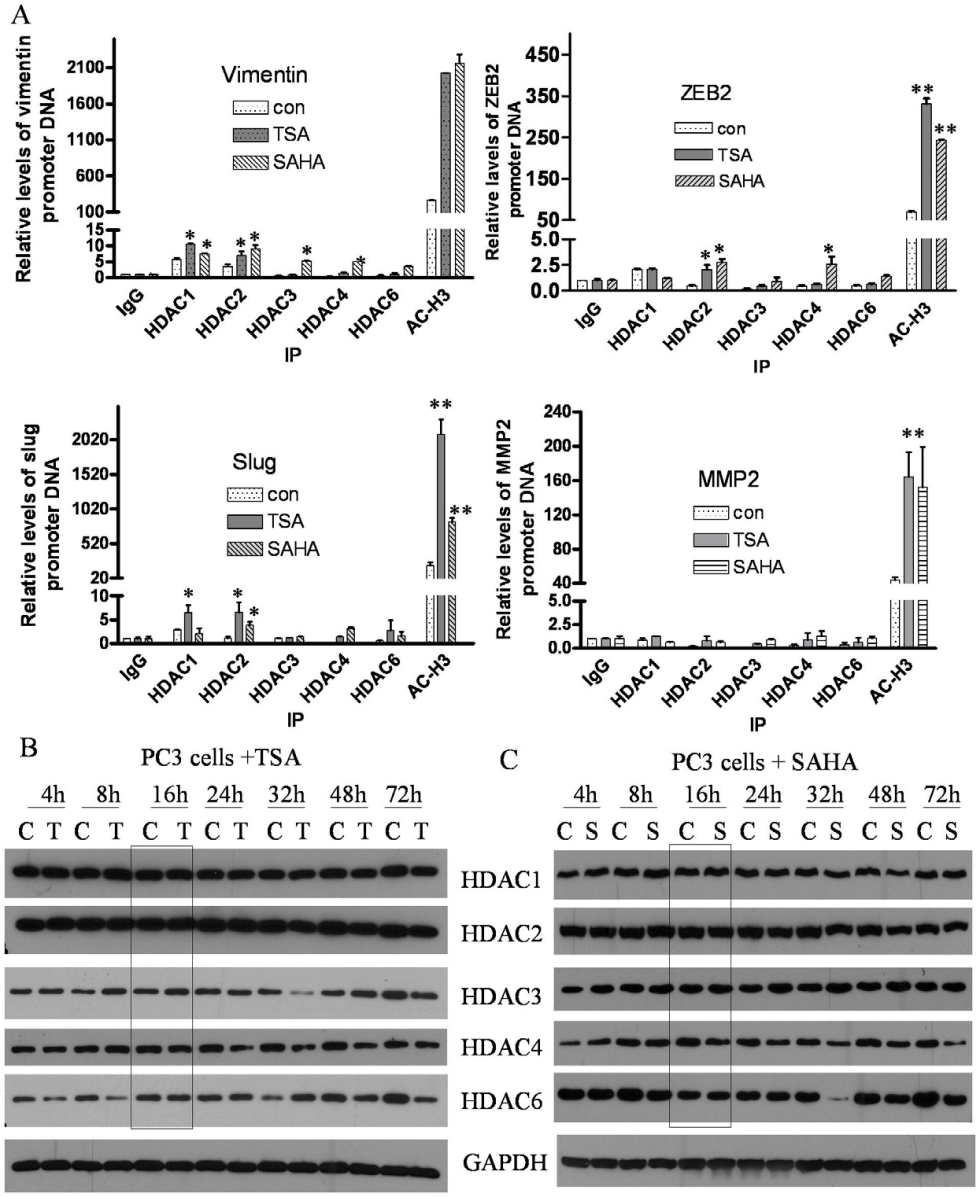
Acetylation of gene promoters by HDACIs was responsible for enhanced expression of these genes. (A) CHIP assay was conducted to determine the binding of HDACs and acetylation status of histone 3 on the promoters of EMT related factors including vimentin, ZEB1, Slug and MMP2 in the PC3 cells treated with TSA and SAHA for 16 h by using HDAC1, HDAC2, HDAC3, HDAC4, HDAC6 and Ac-H3 antibody (*, p<0.05; **, p<0.01). (B) and (C) PC3 cells were treated with TSA and SAHA, and subsequent Western blot analysis showed that the expression of HDAC1, HDAC2, HDAC3, HDAC4 and HDAC6 was not changed at 16 h treatment with TSA or SAHA. (C: DMSO control; T: TSA; S: SAHA).

### HDACIs Increased Expression of Transcription Factors and Mesenchymal Markers

EMT related transcription factors play critical roles in regulating the expression of epithelial and mesenchymal markers. PC3 cells treated with TSA significantly up-regulated mRNA expression of ZEB1, ZEB2, Slug, Twist and snail1 after 24 h treatment (Fig. 2A), which were associated with increased expression of N-cadherin and vimentin. It is known that cells with EMT phenotype acquire increased cell motility, and we found that TSA treatment led to increased expression of MMP2 (Fig. 2A), which was associated with increased cell migration and invasion. To confirm whether HDACIs function as inducers of EMT, we choose another HDACI, SAHA, which has been approved by FDA for its clinical utility. Interestingly, SAHA treatment also led to the acquisition of EMT phenotype consistent with increased EMT marker expression (Fig. 2B). PC3 cells are androgen receptor (AR) negative PCa cell line. In order to assess whether HDACIs could also induce EMT in AR positive PCa cells (such as LNCaP cells), we treated the LNCaP cells with SAHA and we found increased expression of ZEB1 and Slug mRNA as early as 8 h of treatment and it was further increased after 16 h of treatment. The expression was slightly reduced but still it was significantly higher in 400 nM TSA treated group compared to control (Fig. 3A, upper panel). The expression of vimentin mRNA was also increased after 16 h of treatment and sustained high level at 24 h, which was consistent with increased protein level after 24 h treatment with TSA (Fig. 3B). However, N-cadherin mRNA levels were increased as early as 8 h treatment. To determine whether altered mRNA expression is associated with the status of chromatin acetylation, we tested the levels of acetylated-histone 3 (Ac-H3) in cells treated with 400 nM TSA. LNCaP cells treated with TSA showed significantly increased Ac-H3 within 8 h of treatment but decreased slightly after 16 h (Fig. 3B), which is consistent with the expression of ZEB1 and Slug, while increased protein expression of vimetin and Fibronectin was observed after 24 h treatment (Fig. 3B). These results suggest that ZEB1 and Slug are involved in the regulation of vimetin and Fibronectin. In contrast, PC3 cells treated with TSA displayed high levels of Ac-H3 from 4 h to 32 h treatment concomitant with increased expression of ZEB1, while enhanced expression of vimentin protein was observed after 16 h treatment with TSA (Fig. 3C). Similar results were also observed in SAHA treated LNCaP and PC3 cells (Fig. 4A, B and C). These results strongly suggest that HDACIs are potent inducers of EMT due to altered acetylation status, consistent with increased expression of EMT markers.

**Figure 7:**
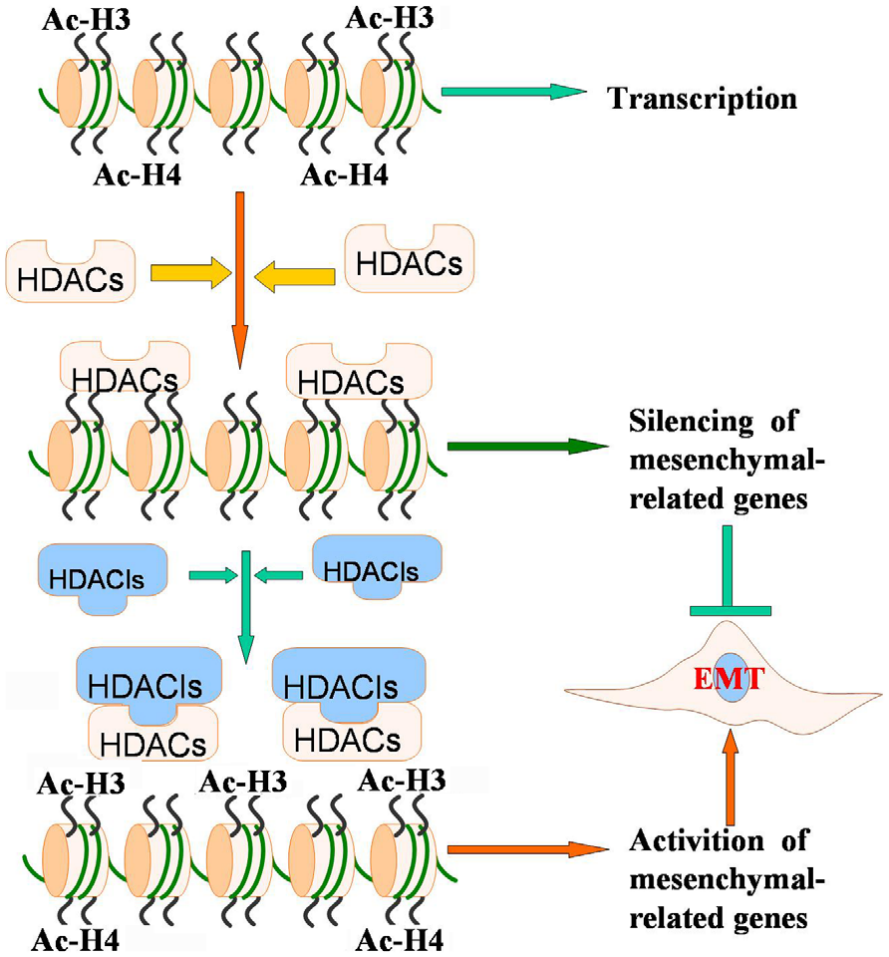
Chromatin remodeling by HDACIs. Recruitment of HDACs by different factors results in deacetylation of histone, leading to gene silence including the silencing of EMT-related genes. Administration of HDACIs induce acetylation of histone, which could activate mesenchymal-related gene expression, resulting in the acquisition of EMT.

### HDACIs Induced Expression of Stem Cell Markers and Increased Cell Motility

The results from Western blot analysis showed that higher doses of TSA (400 nM) and SAHA (5 µM) could promote acetylation of histone and thereby regulates the expression of EMT related factors (Fig. 3B and C: Fig. 4B and C). A dose kinetic study showed that as little as 0.1 µM TSA or SAHA could cause acetylation of histone 3 (increased Ac-H3) in a dose-dependent manner in PC3 cells, which was consistent with enhanced expression of vimentin and N-cadherin (Fig. 5A). However, E-cadherin expression was not significantly changed. The cells with EMT phenotype have been demonstrated to be the source of cancer stem-like cells CSLCs) [10], [11], [16]. To address this issue, PC3 PDGF-D cells with EMT features were treated with TSA at lower dose of range from 50 nM to 200 nM. The results from Western blot analysis showed that the treatment of cells with TSA led to increased expression of stem cell markers such as Sox2 and Nanog in a dose dependent manner (Fig. 5B). Although PC3 PDGF-D cells displayed high levels of vimentin, the expression of vimentin was further increased in TSA treated cells (Fig. 5B). Cells with EMT and/or CSLC signatures showed increased cell motility. Cell detachment from the primary site is a prerequisite for cell migration, and interestingly, TSA or SAHA treatment significantly promoted detachment of PC3 cells from culture plate surface (Fig. 5C), which was consistent with increased trends of cell migration (Fig. 5D).

### Hyper-acetylation of Promoters by HDACIs was Responsible for Regulation of EMT Related Factors

The levels of histone acetylation play crucial roles in chromatin remodeling leading to the regulation of gene transcription. Acetylation of lysine in histone tails is associated with gene-transcription activation owing to a more relaxed chromatin state, which promotes the accessibility of the transcription complexes to the transcription start site. In order to assess whether TSA and SAHA treatment could affect the state of acetylation in histone 3 on the promoters of EMT related factors, we performed CHIP assay. As shown in Fig. 6A, the amount of Ac-H3 associated with proximal promoters of vimentin, ZEB2, Slug and MMP2 was increased significantly in PC3 cells treated with TSA and SAHA compared to DMSO treated control cells. These results suggest that HDACIs induced hyper-acetylation in histone 3 on promoters of vimentin, ZEB2, Slug and MMP2 is mechanistically responsible for enhanced expression of these factors. Acetylation status is depended on the activity of HDACs and their expression levels. Thus, the levels of HDACs were examined in cells treated with TSA or SAHA by Western blot analysis, and as shown in Fig. 6B and C, neither TSA nor SAHA treatment for 16 h showed any change in the expression of HDACs. However, we found that the amount of HDAC1 associated with promoter of vimentin and Slug was higher in TSA treated cells compared to control cells. Moreover, the amount of HDAC2 associated with the promoter of vimentin, ZEB1 and Slug was higher in both of TSA and SAHA treated cells compared to control cells (Fig. 6A). These results suggest that HDAC inhibiters lead to hyper-acetylation in histone 3 on gene promoters by repressing the activity but not reducing amount of HDACs on promoter of target genes such as vimentin, ZEB1, Slug and MMP2, resulting in EMT signatures in prostate cancer cell lines (Fig. 7). Thus, hyper-acetylation of promoters by HDACIs could be responsible for regulation of EMT related factors.

## Discussion

The histone deacetylase inhibitors (HDACIs) have been shown to be promising reagents for the treatment of a number of hematological malignancies including cutaneous T-cell lymphoma [27]–[29] and peripheral T-cell lymphoma in clinical trials [30]. However, clinical trials with the HDACIs in solid tumors have been disappointing, which is in part could be due to the acquisition of resistance to HDACIs [3]. Moreover, mechanisms of resistance have not been fully elucidated. In the current study, we found that HDACIs such as TSA and SAHA could induce epithelial-to-mesenchymal transition (EMT) phenotype and acquired cancer stem-like cell (CSLC) characteristics, which have been known to contribute to drug-resistant, cancer recurrence and metastasis [16], [17]. Our results are consistent with the findings showing that TSA treatment led to increased expression of vementin, which was mediated through promoting acetylation of proximal promoter region of vimentin gene via relieving the repression complex including ZBP-89 and HDAC1 [31]. Inhibition of vimentin expression has been shown to change prostate cancer cell morphology leading to reduced tumor growth *in vivo* [32], and thus increased expression of vimentin is expected to have aggressive tumor cell behavior as supported by our results presented in this manuscript.

A recent study has shown that SAHA could induce EMT in human endometrial adenocarcinoma cell line (Ishikawa cells), which was consistent with up-regulation of N-cadherin and vimentin with concomitant down-regulation of E-cadherin and increased cell motility [33]. Several other studies created controversies based on results showing that HDACIs could actually inhibit EMT by up-regulation of E-cadherin [34]–[39]. Yoshikawa et al. have shown that higher dose of TSA (1000 nM) could prevent changes in cells morphology induced by TGFβ-1 in human renal epithelial cells. However, no effect on cell morphology induced by TGFβ-1 was seen at 100 nM TSA but some mild effect was seen with 300 nM of TSA treatment, which was associated with increased E-cadherin expression. However, increased expression of vimentin and N-cadherin are the hallmarks of EMT, which was not tested in this study [39]. Another study showed that TSA could up-regulate the expression of E-cadherin through activation of E-cadherin promoter via acetylation of histone on E-cadherin promoter, concomitant with increased expression of snail, Slug and Twist1, but mesenchymal markers such as vimentin and N-cadherin were not investigated [38].

Transcription factors such as snail, Slug and Twist1 not only inhibit E-cadherin expression, but also lead to increased expression of mesenchymal markers such as vimentin and N-cadherin via activation of promoters of the target genes. These results suggest that HADCIs are believed to increase the expression of mesenchymal-related factors, while the effect of HADCIs on E-cadherin could be depended on the acetylation status of E-cadherin promoter and the doses of HADCIs used. In our study, TSA or SAHA treatment induced the expression of mesenchamal-related factors including transcription factors and mesenchymal markers, while the expression of E-cadherin was not changed or decreased in PC3 and LNCaP cells. Co-expression of epithelial and mesenchymal markers has been previously observed; for example, both epithelial and mesenchymal markers has been documented in circulating tumor cells from prostate cancer and breast cancer patients [40]. More importantly, circulating tumor cells not only exhibited EMT phenotype but also expressed stem cell markers [40]–[43]. These findings are consistent with our results that HDACIs lead to the acquisition of EMT and CSLC characteristics in PCa cells.

Mounting evidence has shown that the cells with EMT phenotype display stem cell features. As expected, our results showed that treatment of PC3 PDGF-D cells (EMT phenotypic cells) with TSA not only enhanced the expression of vimentin but also up-regulated the expression of stem cell markers including Sox2 and Nanog in a dose dependent manner. These results are consistent with findings showing that valproic acid (VPA), a histone deacetylase (HDAC) inhibitor, could induce the expression of Sox2, Oct4 and Nanog in skeletal myoblasts purified from young male Oct3/4-GFP+ transgenic mouse [44]. Recent studies have also shown that co-expression of pluripotency markers such as Oct4, Sox2, Nanog and Lin28 can reprogram somatic cells into pluripotent embryonic stem-like cells [45]. These results suggest that combined expression of stem cell-associated factors could be associated with an undifferentiated state in cancer cells.

Gu et al. has shown that immortalization of cell lines derived from human PCa specimen showing epithelial phenotype by hTERT showed the expression of embryonic stem cell markers such as Oct4, Nonag, and Sox2 [46]. Interestingly, HDACIs such as TSA, SAHA and VPA not only increased the reprogramming efficiencies during induction of iPS (inducible pluripotency stem cells) cells by co-expression of pluripotency factors [47], but also facilitates a reduction in the number of reprogramming factors [48], [49]. These results suggest that HDACIs regulate the function of Oct4, Nonag, and Sox2 during the induction of embryonic stem cell signatures mediated by the induction of endogenous expression of Oct4, Nonag, and Sox2. Moreover, over-expression of Oct4, Sox2 and Nanog has been found in poorly differentiated tumors [50], which could be associated with tumor progression and metastasis. In summary, our results clearly suggest that HDACIs could induce EMT and CSLC characteristics in PCa cells, and thus the clinical utility of HDACIs in PCa should proceed with a great caution. However, reversal of EMT to MET phenotype by novel agents prior to HDACIs could be useful for the treatment of solid tumors especially PCa, and thus further investigations along this line is warranted.
